# Society Meetings

**Published:** 1908-06

**Authors:** 


### SOCIETY MEETINGS.

THE International Congress on Tuberculosis will convene at Washington, D.C., September 21 and continue until October 12, 1908. The President of the United States has accepted the presidency of the congress and discusses the importance of the subject in a somewhat extended letter of acceptance. We shall print the letter in a future number.

THE Fifth Pan-American Medical Congress will be held at Guatemala, C. A., August 5-8, 1908.

THE Buffalo Academy of Medicine held meetings during April and May as follows:

A special meeting was held Tuesday evening, April 28, to consider the question of incorporating the Academy and the question of a home. The program was furnished by the Section on Obstetrics and Gynecology: My obstetrical experience, J. W. Grosvenor.

*Section on Surgery.*—Tuesday evening, May 5. Program: Patients with enlargement of the prostate who should not be operated upon by prostatectomy, Paul Thorndike, Professor Genitourinary Diseases, Harvard Medical School, Boston.

*Section on Medicine.*—Tuesday evening, May 12. Program: Bacterial Vaccines: (*a*) The vaccines in their application to the ordinary 'tyogenic case, Norman K. MacLeod; (*b*) The vaccines in their relation to erysipelas, W. G. Ross, Toronto, Ont.

*Section on Pathology.*—Tuesday evening, May 19. A chairman and a secretary of the section for the ensuing year were elected at this meeting. Program: Care of the municipal milk supply. Lantern slide demonstration, George W. Goler, Commissioner of Health, Rochester, N. Y.

THE Rochester Academy of Medicine held meetings during May as follows:

*Section on General Medicine.*—Wednesday evening, May 6.

Program: Diagnosis of tuberculosis, Charles D. Young; Treatment of tuberculosis, George W. Goler.

*Section on Surgery.*—Wednesday evening, May 13. Program: "Honor to Whom Honor is Due"—a tribute to the late Dr. Henry Koch, E. Wood Ruggles.

